# Knowledge and attitude of pregnant women in the Kingdom of Saudi Arabia toward Noninvasive prenatal testing: A single center study

**DOI:** 10.1002/mgg3.1960

**Published:** 2022-04-28

**Authors:** Maaged A. Akiel, Mohamud S. Mohamud, Emad M. Masuadi, Hassan S. Alamri

**Affiliations:** ^1^ Department of Clinical Laboratory Sciences, College of Applied Medical Sciences King Saud bin Abdulaziz University for Health Sciences (KSAU‐HS) Riyadh Kingdom of Saudi Arabia; ^2^ King Abdullah International Medical Research Center (KAIMRC) Riyadh Kingdom of Saudi Arabia; ^3^ Department of Human and Molecular Genetics Virginia Commonwealth University Virginia USA; ^4^ Research Unit, College of Medicine King Saud bin Abdulaziz University for Health Sciences (KSAU‐HS) Riyadh Kingdom of Saudi Arabia

**Keywords:** attitudes, decision‐making, knowledge, prenatal diagnosis

## Abstract

**Background:**

Noninvasive prenatal testing (NIPT) is a screening tool for chromosomal aneuploidies. Prior knowledge of NIPT is an inherent factor in the decision‐making process. We assessed the knowledge and attitude of pregnant women related to prenatal testing with a particular focus on NIPT.

**Methods:**

A prospective cross‐sectional study, using a culturally validated questionnaire, was conducted with 342 pregnant women of whom 74.9% consented for prenatal screening. Mean age and gestational weeks ± standard deviation was 31 ± 5 and 26 ± 11, respectively.

**Results:**

A positive/very positive attitude was observed to ultrasound, followed by FCT, NIPT, and lastly to CVS. More than half of the participants (56.1%) had no previous knowledge of NIPT. A reaching significance association was detected between education and knowledge of NIPT. Significant association was detected between risk for aneuploidy and knowledge of NIPT. The majority (74%) indicated their willingness to perform the test. The effect and value of society on the pregnant women to make a decision regarding NIPT was negligible.

**Conclusion:**

The pregnant women in the current study displayed a lack of knowledge and awareness regarding prenatal screening, particularly the NIPT. We recommend that pregnant women receive adequate counseling regarding prenatal screening to increase their awareness and knowledge of prenatal testing, including NIPT.

## Graphical abstract

We report reduced knowledge and awareness of pregnant women towards prenatal screening with particular focus on NIPT. We conclude from this study that pregnant women are not adequately counseled during their visit to the Obstetrics and Gynecology clinic.

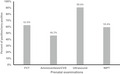

## 1 INTRODUCTION

Chromosomal abnormalities are considered a major cause of stillbirth and an increased risk of a miscarriage (Wilkins‐Haug, 2020), and are dichotomized in numerical and structural abnormalities (Zhu et al., 2016). Structural abnormalities are defects in the structure of the chromatin, which include insertions/deletions (indels), duplications, inversions, and ring formations (Kaser, 2018). Numerical abnormalities are alterations in the number of chromosomes that alter the gene dosage (Kaser, 2018). The most common aneuploidy is Down syndrome (DS), a congenital disorder characterized by an error in the maternal meiotic cell division called “non‐disjunction.” As a result, a chromosomal gain in chromosome 21 (Trisomy 21) occurs (Kaser, 2018). Globally, the frequency of Down syndrome is 1:800 live births compared to 6.6:10,000 live births in the Kingdom of Saudi Arabia (AlSalloum et al., 2015; Bull, 2020). A miscarriage/stillbirth was reported in 10% of pregnancies with Trisomy 21 (Won et al., 2005). Viable births with Trisomy 21 suffer from several abnormalities, including cardiac, gastrointestinal, craniofacial, and orofacial defects. Such defects can be a burden to the family and society (AlSarheed, 2015; Bull, 2020). Among other chromosome abnormalities are Sex Chromosome Aneuploidies (SCAs), which include monosomy X (Turner syndrome), 47, XXX (Trisomy X syndrome), 47, XXY (Klinefelter syndrome), 47, XYY syndrome, and 48, XXYY syndrome, reviewed in (Skuse et al., 2018). Variation of incidence rate among SCAs is observed. For example, Monosomy X and Klinefelter syndrome have an incidence rate of 1:2500, 1:750, respectively (Skuse et al., 2018). On the other hand, Trisomy X syndrome and 48, XXYY are seen in 1:20,000 and 1:1000 live births, respectively (Skuse et al., 2018). Additionally, phenotypic variation among SCAs was recorded. Turner syndrome live birth tend to have increased learning difficulties compared to mild learning difficulties in Trisomy X live births (Skuse et al., 2018) When rate of miscarriage is examined, SCA tend to score lower miscarriage rates. For example, monosomy X have low risk of miscarriage, between 6% and 16% of 45, X positive pregnancies (Gug et al., 2019; Ljunger et al., 2005). Similarly, miscarriages in 47,XXY positive pregnancies were as low as 3.4% (Ljunger et al., 2005). However, a high percentage of miscarriages were reported in Trisomy 13 and 18 at week 9–14 of gestation, 40% and 70%, respectively (Cavadino & Morris, 2017). The risk of miscarriage is also variable among SCA in which a recent study found that SCA are more common in women that are younger or equal to 35 years of age (Gu et al., 2021). Therefore, the risk of miscarriage in chromosomal abnormalities should be accurately calculated and explained to pregnant women to provide the best available prenatal care. During pregnancy, cell‐free DNA of the fetus sheds from the syncytiotrophoblast layer of the placenta because of the cellular turnover (Alberry et al., 2007; Flori et al., 2004). Consequently, the amount of fetal cell‐free DNA in the maternal plasma increases proportionally with gestational age (Lo et al., 1997; Shaw et al., 2020; Sun et al., 2018). The use of placental cell‐free DNA for screening purposes was first introduced by the American College of Obstetricians and Gynecologists and the Society for Maternal‐Fetal Medicine in 2011 for women with an increased risk of aneuploidy (“Committee Opinion No. 640,” 2015). Subsequently, policies were drafted to regulate the reporting of noninvasive prenatal testing (NIPT) and counseling of pregnant women in western countries (Dondorp et al., 2015). According to the recommendations set by the Prenatal Screening Committee at the International Society for Prenatal Diagnosis (ISPD), all pregnant women should use NIPT as a primary prenatal screening tool (Benn et al., 2015). The advantages of NIPT compared with invasive prenatal screening include ease of sample collection and lack of risk of miscarriage as it only requires small amount of maternal blood (Spencer et al., 2020). The increased sensitivity of next generation sequencing technologies facilitated the use of NIPT for screening as early as 10 weeks of pregnancy compared to 13 and 15 weeks for Chorionic villus sampling (CVS) and Amniocentesis (Spencer et al., 2020). NIPT was found to be optimal for screening of aneuploidies such as Trisomy 21, 18, and 13 (Spencer et al., 2020). However, the accuracy of screening for aneuploidies of sex‐chromosomes was not acceptable (Deng et al., 2019; Kornman et al., 2018; Lu et al., 2021). In the Kingdom of Saudi Arabia, the Ministry of Health launched two national programs for screening of genetic disorders, the Premarital Screening Program and the Newborn Screening Program (Gosadi, 2019). The premarital screening program aims to screen couples at risk for frequent Mendelian disorders in the region, primarily sickle cell anemia and Thalassemia (Gosadi, 2019). The newborn screening program focuses on the inborn errors of metabolism (Gosadi, 2019). A program for prenatal screening and counseling of pregnant women in the Kingdom of Saudi Arabia is lacking (Ne et al., 2017). Prenatal screening, mainly ultrasound, is only offered at the OB/GYN clinic in Saudi Arabia. Additionally, prenatal counseling of pregnant women is only offered at specific locations to high‐risk women, defined as pregnant women with previous history of aneuploidies (Balobaid et al., 2016). In a previous study, 920 senior college students (mainly females) displayed a lack of knowledge when asked about the risk assessment of genetic disorders and prenatal screening (Olwi et al., 2016). Adequate knowledge of the genetic risk to congenital abnormalities positively influences decisions to undergo genetic testing (Etchegary et al., 2010). A lack of knowledge of genetic testing would result in a negative attitude to prenatal screening and would affect the ability of pregnant women to make an informed decision regarding prenatal screening. Factors affecting the knowledge of NIPT are attributed to the tendency of self‐learning, level of education, and the health care provider (Olwi et al., 2016; Wittman et al., 2016). Increasing the knowledge of women of prenatal screening would positively increase their awareness and ability to make an informed medical decision for testing. In the Kingdom of Saudi Arabia, the choice of terminating a pregnancy is only allowed under a strict regulation. Termination of pregnancy is allowed before the 120th day of conception as this marks the day of fetal ensoulment, according to the Islamic law (Al Aqeel, 2007). The Kingdom of Saudi Arabia follows Islamic laws, and abortion is prohibited after ensoulment as preservation of human life is one of the basic principles of Islamic law (Al Aqeel, 2007). A previous study with the Saudi population found that decisions regarding terminating a pregnancy depend on the severity of the underlying genetic disorder. For example, termination of pregnancy was favored in case of trisomy 13 and 18 (Alsulaiman & Hewison, 2007). NIPT provide an advantage for the community as it can provide answers from the 10th week of pregnancy allowing families to decide regarding the termination of the pregnancy prior to ensoulment (Spencer et al., 2020). Due to the lack of knowledge and awareness of pregnant women regarding prenatal screening in Saudi Arabia, we aimed to assess the knowledge and attitude of pregnant women with a particular focus on NIPT. We used a culturally validated questionnaire to the Saudi Arabian community (Akiel et al., 2020). We surveyed 342 pregnant women who attended the Obstetrics and Gynecology (OB/GYN) clinic at King Abdulaziz Medical City in Riyadh, Kingdom of Saudi Arabia. The questionnaire included patient characteristics such as age, educational level, number of miscarriages, previous experience with congenital abnormalities, and type of performed prenatal examination tests. Other questions included attitude and knowledge related to prenatal testing, with a focus on NIPT and the factors influencing the decision to perform the NIPT.

## 2 METHODS

### 2.1 Data collection and questionnaire distribution

A prospective cross‐sectional study was conducted with pregnant women attending the OB/GYN clinic at King Abdulaziz Medical City, Riyadh, Kingdom of Saudi Arabia from December 2018 to April 2019, using a questionnaire validated for the Saudi Arabian community (Akiel et al., 2020). All pregnant Arabic women who attended the OB/GYN clinic were included. The clinic nurses distributed the printed questionnaires after their regular consultation. The background information regarding prenatal screening were attached to the questionnaire. The questionnaires were collected at the end of the week. The details regarding the questionnaire contents were previously described (Akiel et al., 2020). The data collected from the questionnaire were entered electronically in a Microsoft Excel file (Supplementary File S1). Questions with Yes and No answers were recorded as 1 or 0, respectively. Likert‐Scale Questions were recorded from 1 to 5.

### 2.2 Patient characteristics

In total, 342 of 400 pregnant women agreed to participate in this study, with a response rate of 85%. The reasons for rejecting to participate in the study were not clear. The mean age ± standard deviation (SD) as 31 ± 5 years and the mean gestational week ± SD 26 ± 11 weeks. Level of education, number of parity, history of miscarriage, and previous fetal examinations were recorded. Participants were classified as high risk for aneuploidy according to increased maternal age (more than 35 years old) and increased miscarriages (more than 3) or previous history with chromosomal abnormalities (Table 1).

**TABLE 1:** Demographic and prenatal testing history of the study participants

Question	Category	*N*	%
Highest completed education	Elementary	23	6.7
	High School	92	26.9
	University	227	66.4
Number of children	None	75	21.9
	1–2	132	38.6
	≥3	135	39.5
Number of miscarriages	None	197	57.6
	1–2	103	30.1
	≥3	42	12.3
Have any examination been performed on your fetus?	No	86	25.1
	Yes	256	74.9
First trimester‐combined test (FCT)	No	280	82.1
	Yes	61	17.9
Amniocentesis	No	311	91.2
	Yes	30	8.8
Chorionic villus sampling (CVS)	No	331	97.1
	Yes	10	2.9
Ultrasound	No	109	32
	Yes	232	68
^a^High risk for aneuploidy	No	252	73.7
	Yes	90	26.3
Age in years	Mean ± SD	31 ± 5	
Gestational week	Mean ± SD	26 ± 11	

^a^ Risk for aneuploidy was classified according to presence of increased maternal age (>35 years old) and increased miscarriages (>3) or previous history of chromosomal abnormalities.

### 2.3 Statistical analysis

The Statistical Package for Social Sciences (SPSS) version 25 (IBM Corp., Armonk, NY, USA) was used to analyze the data. Continuous variables are expressed as mean ± SD and the categorical variables as a frequency and percentage. A chi‐square test was used to assess the association between the categorical variables. A test was considered significant if the *p*‐value was <0.05.

## 3 RESULTS

### 3.1 The perception of pregnant women of chromosomal aberrations

**FIGURE 1:**
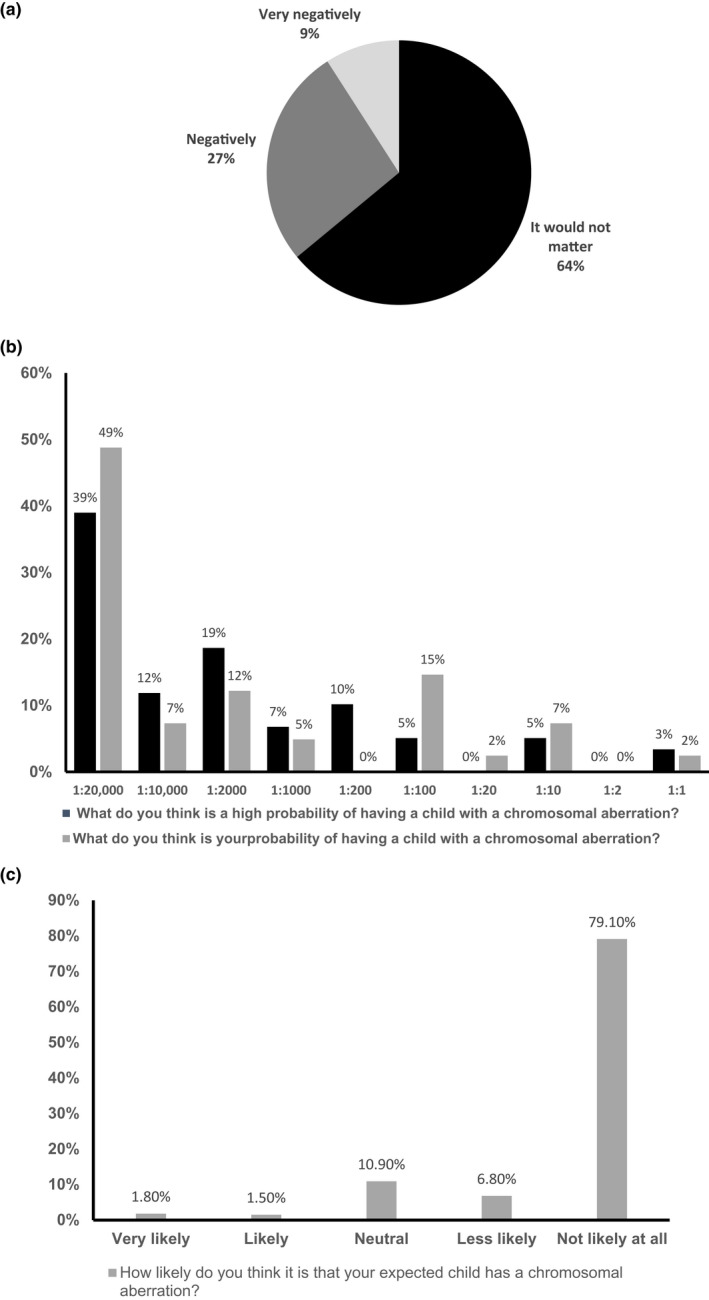
Perception of pregnant women on having a child with chromosomal aberrations. (a) Perception of the sample (*n* = 342) when asked about their reaction upon having a child with chromosomal abnormality, such as Down syndrome. (b) Percentage distribution of the sample (*n* = 342) on what they think is considered a high probability and what their probability is of having a child with chromosomal abnormality, using mathematical expression. (c) Percentage distribution of the sample (*n* = 342) and their likelihood of having a child with chromosomal abnormality using a Likert scale (1: Not likely at all‐ 5: Very likely)

When asked regarding their reaction should they give birth to a child with a chromosomal anomaly, the majority (64%) reported that it would not matter. The rest answered that they would react negatively (27%) and very negatively (9%) (Figure 1a). To assess the knowledge of the probability of a pregnancy with a chromosomal anomaly, we asked the participants to select from a series of ratios the one indicating a high probability. The highest proportion (39%) indicated 1:20,000 as a high probability, 19% 1:2000, 12% 1:10,000, 10% 1:200, 7% 1:1000, and 5% 1:100 and 1:10. Only 3% selected 1:1 as a high probability. When asked what, in their opinion, is their probability of having a child with a chromosomal anomaly, almost half (49%) selected 1:20,000, 15% 1:100, 12% 1:2000, and 7% 1:10,000 and 1:10, Only 5% selected 1:1000 and 2% selected 1:1 and 1:20 (Figure 1b). Similarly, using the same question with a Likert‐scale, the majority (79.1%) indicated that it is not likely at all for them to have a child with a chromosomal abnormality, 10.9% had a neutral response, 6.8% less likely, 1.8% very likely, and only 1.5% likely (Figure 1c).

### 3.2 Attitudes of pregnant women regarding prenatal screening

**FIGURE 2:**
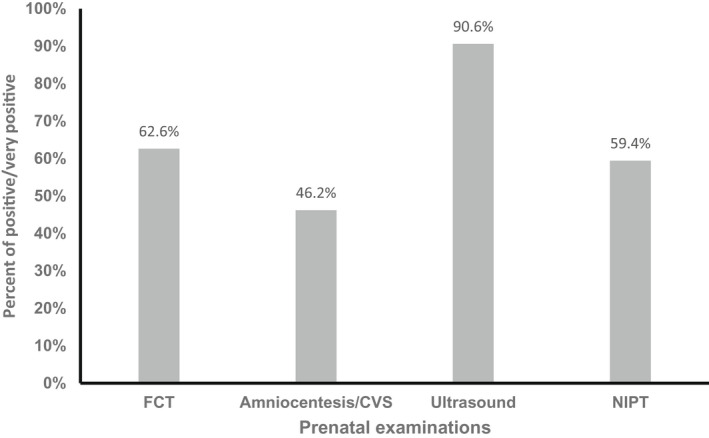
Attitude of pregnant women toward prenatal testings. Percentage distribution of the sample (*n* = 342) who selected an attitude of positive/very positive

To evaluate the attitude of the pregnant women regarding prenatal testing including NIPT, we asked the sample whether they had heard of NIPT before. As expected, more than half of participants (56.1%) had no prior knowledge of NIPT (Table 2). We assessed their perception of prenatal screening as Good/Bad, Frightening/Not frightening, or Calming/Not Calming. Very good was selected by 71.1% of the participants, 27% were Neutral, and 1.2% Good. In terms of fear of prenatal screening, 10.5% indicated Very frightening, 76.6% Neutral, and only 12.9% Not frightening at all. Regarding prenatal screening being calming, 2% selected Calming, 5.2% Not calming at all, and the rest of 76.9% were Neutral (data not shown). The sample's attitude to each of the prenatal screening tests, including NIPT, was measured with a Likert‐scale, starting from very negative (1) to very positive (5). Ultrasound achieved the highest score (90.6%) of a very positive/positive attitude, the First Trimester‐Combined Test (62.6%), NIPT (59.4%), and finally amniocentesis/CVS (46.2%) (Figure 2).

**TABLE 2:** Attitude of pregnant women toward NIPT

Question	*N*	%
Heard of NIPT test?		
No	192	56.1
Yes	150	43.9
Would you like to have the test if available?		
Absolutely not sure	31	9.1
Not sure	13	3.8
Neutral	31	9.1
Sure	14	4.1
Completely sure	253	74.0
If this blood sample would not be covered by the national health insurance, would you be willing to pay by yourself?		
No	146	42.7
Yes	196	57.3
If yes, how much would you be willing to pay?	US$	%
	6024	1.5
	1205	2.5
	603	9.6
	241	23.9
	120	45.8
	60	100

### 3.3 Association between knowledge of NIPT and education level

We measured the association between the patients characteristics and knowledge of NIPT. We performed a chi‐square test on age, educational level, number of miscarriages, previous history with chromosomal abnormalities, and high risk for chromosomal aneuploidies. A reaching significance association was observed between the education level and knowledge of NIPT. The proportion who knew about NIPT was higher in the group with high school education and above. Additionally, we observed significant association between the high‐risk group for aneuploidy and knowledge of NIPT. The level of knowledge also increased in the group with an increased number of miscarriages (≥3) or a previous history of aneuploidies, however the difference was not statistically significant (Table 3).

**TABLE 3:** Association between knowledge of NIPT and patient characteristics

	Heard of NIPT test				
	No		Yes		*p*‐value
	N	%	N	%	
Age					
≤25	34	57.60	25	42.40	0.506
26–30	70	57.90	51	42.10	
31–35	50	59.50	34	40.50	
>36	38	48.70	40	51.30	
Highest completed education					
Elementary	18	78.30	5	21.70	0.063
High school	47	51.10	45	48.90	
University	127	55.90	100	44.10	
Miscarriage					
≤2	172	57.30	128	42.70	0.235
≥3	20	47.60	22	52.40	
Previous experience with congenital abnormalities					
No	183	65.70	140	43.3	0.428
Yes	9	47.4	10	52.6	
High risk for aneuploidy					
No	150	59.5	102	40.5	0.035
Yes	42	46.7	48	53.3	

### 3.4 Willingness to pay for NIPT

To measure the acceptance of the sample to NIPT as a preferential prenatal screening test, we explored whether the sample would choose the test for prenatal screening if available. The majority (74%) indicated completely sure, however, a small proportion (9.1%) were absolutely not sure about their selection of the NIPT for prenatal screening. A small proportion (3.8%) indicated not sure, neutral (9.1%), and sure (4.1%) (Table 2). When asked if they would be willing to pay for NIPT if the health insurance company refused, almost half (57.3%) would pay. The amount they are willing to pay ranged from 60 to 120 US$, which is 225 to 450 Saudi Riyals (Table 2).

### 3.5 Desired information from the NIPT

**FIGURE 3:**
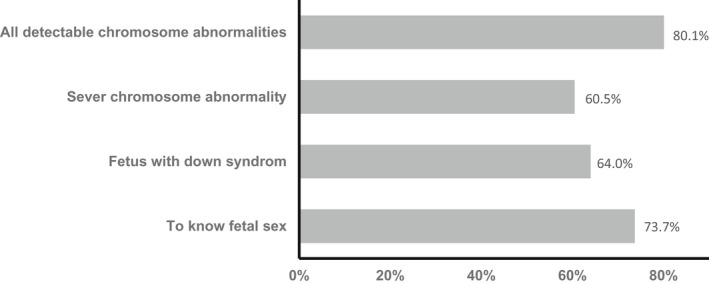
Desired information to be included in the NIPT. Percentage of desired information selected by the sample (*n* = 342). Participants were allowed to pick multiple answers

Exploring the type of information that the sample would require from the NIPT, the sample had to respond with YES or NO to several options that they would want in the NIPT report (multiple selections were allowed). The options included fetal gender, Down syndrome, severe chromosome abnormalities and all detectable chromosomal abnormalities (Figure 3). The majority (80.1%) expected that the NIPT should screen for all detectable chromosomal anomalies. Surprisingly, 73.7% were interested in knowing the fetal gender. The rest of the options were almost equally distributed, severe chromosomal abnormalities (60.5%) and fetus with Down syndrome (64%) (Figure 3).

### 3.6 Factors affecting the decision to perform NIPT

**FIGURE 4:**
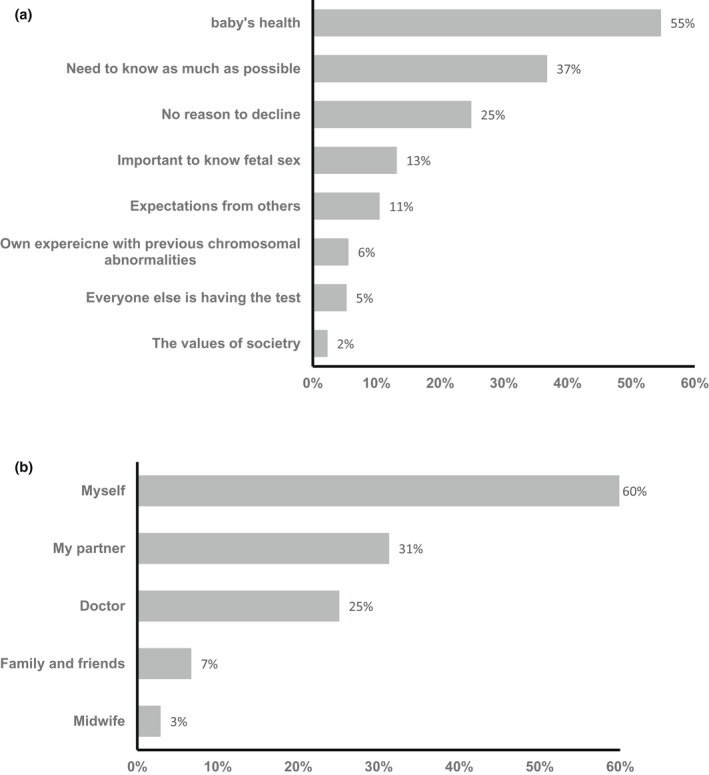
Factors affecting the decision to undergo NIPT chromosomal examinations. (a) Percentages of factors affecting the decision of 342 surveyed pregnant women to perform NIPT. (b) Percentages were distributed from highest to lowest. Percentages of individuals that can affect the decision of 342 surveyed pregnant women to perform NIPT. Percentages were distributed from highest to lowest

Regarding the factors that influence the decision to perform a NIPT, the sample could choose from a series of options (multiple selections were allowed). The highest proportion indicated the baby's health, which was statistically significant. Curiosity of knowing as much as possible about the baby was selected by 37%, and no reason to decline (25%). The fetal gender, expectations from others and own previous experiences with chromosomal abnormalities received 13%, 11%, and 6%, respectively. Notably, only 2% chose the value to the society and 5% everyone else is doing the test (Figure 4a). Influential persons in the decision‐making process were indicated as myself (60%), my husband (31%), the doctor (25%), family and friends (7%), and lastly the midwife (3%) (Figure 4b).

## 4 DISCUSSION

Lack of knowledge, awareness, and counseling of pregnant women in the Kingdom of Saudi Arabia of prenatal screening, particularly regarding NIPT, can affect their selection of a suitable prenatal screening test. We report an assessment of their knowledge, awareness, and attitude, using the previously validated NIPT‐related questionnaire (Akiel et al., 2020). The participants of the current study included pregnant women who attended the OB/GYN clinic at King Abdulaziz Medical City in Riyadh, Kingdom of Saudi Arabia. The majority of the sample did not perform any advanced prenatal screening such as FCT, CVS, amniocentesis, or NIPT (Table 1), indicating that they were not aware or adequately counseled regarding the availability of the prenatal screening tests. A second possibility is that during pregnancy, FCT tests became routine tests and the pregnant women may not have known that a screening test was done until the result was positive. In addition, the lack of knowledge and awareness may also be due to the fact that FCT, CVS, amniocentesis, or NIPT are not usually performed unless a positive result of a screening test requires confirmation, for example an ultrasound. Our findings are in contrast to literature reporting pregnant women as more aware regarding prenatal screening during their visits to the OB/GYN (Quaresima et al., 2020; Yang et al., 2021). Conducting prenatal screening, such as FCT, CVS, and amniocentesis improves the detection of congenital anomalies. We recommend educating pregnant women about the type of tests performed during their prenatal visits. This is required to increase their knowledge and awareness of the availability of the multiple options for prenatal screening (Braz et al., 2018). The aim of the pre‐marriage national program in the Kingdom of Saudi Arabia is to screen couples carrying sickle cell and thalassemia alleles (Gosadi, 2019). This is due to the increased prevalence of these Mendelian disorders in the region as a result of consanguineous marriages (Gosadi, 2019). Consanguinity increases the inbreeding coefficient and therefore the chance that a recessive allele would be shared by the couple increases (Hamamy, 2012), increasing the risk of Mendelian recessive disorders. The program does not include prenatal screening or counseling of pregnant women regarding prenatal screening tests available to them (Gosadi, 2019). A consultation with a genetic counselor, in addition to their visit to the OB/GYN is limited, as it is only offered to a specific population of pregnant women in a limited location in the Kingdom of Saudi Arabia (Balobaid et al., 2016). A previous study in the United States reported that pregnant women spent 45–60 minutes with a genetic counselor, in addition to their regular OB/GYN visit. During this visit, the genetic counselor invested more time explaining on the availability and limitations of available prenatal screening tests than the OB/GYN (Wittman et al., 2016). As a result, this practice significantly increased the knowledge and awareness of pregnant women regarding prenatal screening (Wittman et al., 2016). The vast majority of prenatal screening in the Kingdom of Saudi Arabia are performed at the OB/GYN clinic. As a result, it may not be sufficient to increase the knowledge and awareness of prenatal screening as a result of the limited time to spend with the OB/GYN, due to the large volume of visitations OB/GYNS receive daily. We believe that incorporating a similar practice to the United States in which pregnant women are seen separately by a genetic counselor is feasible as the number of genetic counselors in Saudi Arabia are increasing due to increased number of genetic counseling training programs in the region (Qari et al., 2013). A limitation to this approach is that we can think of covering the demand for different regions in the country, however, with the newly launched government transformation programs which include initiatives for electronic health and telecom health, we believe that remote counseling sessions would increase the geographical area of patient coverage using available advanced telecommunication technologies (Chowdhury et al., 2021). As a result, this would increase the awareness of pregnant women in the Kingdom of Saudi Arabia. The majority (64%) of the sample in the current study replied that they would accept the outcome, if diagnosed with aneuploidy (Figure 1a), choosing to continue with the pregnancy rather than termination. The underpinning is the inherent belief of the basic principle of Islamic law regarding the preservation of human life. Similarly, 57.1% of Chinese pregnant women reported that they would accept the outcome and not terminate the pregnancy (Lau, Chan, et al., 2012). When asked about their perception regarding what a high probability is, 39% indicated a 1:20,000 chance as a high probability. This indicate that a good proportion of the sample lacked basic probability knowledge and they need to be taught about the probability of chromosomal anomalies during their visits to the OB/GYN clinic. The sample was confident that they are at a low risk of delivering a child with a chromosomal abnormality (Figure 1b,c). This could be due to their young age and the belief that chromosomal aberrations are caused by a high maternal age or reassurance from a negative ultrasound result (Table 1) and (Figure 2). Although maternal age was associated with chromosomal aneuploidies, a causative link to aneuploidy was not established (Callaway et al., 2005). These findings emphasize the lack of knowledge in the sample regarding the fundamentals of genetic disorders. The availability of NIPT was not known to 56.1% of the sample. The group with a higher education were more aware of the availability of NIPT, than the group with elementary education, supported by literature (Wittman et al., 2016; Yang et al., 2021). This suggests that the level of education plays a significant role in acquiring new knowledge. Women with a higher education are probably more literate, in terms of searching the Internet for information. They also may have improved communication with their healthcare provider and acquire information regarding screening from the OB/GYN. We also found increased level of knowledge regarding NIPT in the high‐risk group for aneuploidy which include participants with increased maternal age and increased history of miscarriage or previous history with chromosomal aneuploidies. Since that prenatal counseling is only performed to high‐risk pregnant women in the Kingdom of Saudi Arabia, we can assume that the observed increase in level of knowledge of NIPT in the high‐risk group in our sample is due their previous experience with genetic counselors and or self‐learning. When asked about NIPT, the majority (74%) of the current sample would do the test if it was available at the OB/GYN clinic. The majority were willing to pay the expenses if not covered by the insurance. This indicates that the benefits of NIPT and the reassurance obtained, outweigh the cost. The sample was comfortable to select the NIPT for prenatal screening due to the ease of sample collection and elimination of the risk of miscarriage. We believe that to be able to include all levels of socioeconomic status, the market price for the NIPT should be less than 450 Saudi Riyals (120US$). It should provide screening for frequent chromosomal aneuploidies, such as Trisomy 13, 18, and 21, for which NIPT can screen with high sensitivity and specificity (Gekas et al., 2014; Gil et al., 2014; Lau, Chen, et al., 2012). The determination of fetal gender should be included as this was highly selected by the sample in our study. The responsibility of the pregnant women for the good health of the future baby is an important factor in the decision‐making to perform the NIPT (Figure 4a). This sense of responsibility for doing the right thing appears to be influenced by the women themselves as 60% indicated myself as a factor that influence making a decision. The other factors influencing the decision regarding NIPT included the partner (31%) and the doctor (25%). Collectively, the influence of the partner and the clinical practitioner have an important impact on the pregnant women's acceptance or rejection of the test (Figure 4). Literature from western countries indicated that the relational factors affecting the decision‐making regarding prenatal screening included the partner, family, and social influence from the maternity unit (Di Mattei et al., 2021). Our findings are similar regarding the impact of the partner and interaction with the clinical practitioner on decision‐making. However, the family and society does not seem to impact the decision‐making for NIPT testing. This indicate that there are sociocultural differences between societies that would require individualized counseling according to the cultural construct. The finding that the partner and the doctor have an influence over prenatal testing, support our recommendation of including the partners in counseling sessions related to prenatal screening. The OB/GYN practitioner should be trained about how to adequately deliver counseling sessions to the visiting couple. Another option would be to refer couples to certified genetic counselors during their visits to the OB/GYN.

The limitations of our study includes the limited sample size and recruitment from a single center. We did not stratify the sample before and after the visit to the OB/GYN or asked the participant whether the OB/GYN provided sufficient time for explanation regarding the available prenatal screening tests and the level of knowledge before and after the visit was not analyzed. We would like to point out that the reason behind this is that our questionnaire was previously validated for the intention to assess general knowledge of NIPT among pregnant women visiting OB/GYN clinic, we preferred not to perform further modification to the questionnaire in the current study because this will affect internal consistency of questionnaire questions. Findings from our study provide an assessment of the knowledge, attitude and awareness of pregnant women attending the OB/GYN clinic at King Abdulaziz Medical City in Riyadh, Kingdom of Saudi Arabia, one of the largest centers in the Middle East. We conclude that our sample lacked knowledge and awareness regarding prenatal screening, including the NIPT. Moreover, we observed increased knowledge of NIPT among high‐risk pregnant women.

## CONFLICT OF INTEREST

Authors declare no conflict of interest.

## AUTHOR CONTRIBUTIONS

Maaged A. Akiel participated in the conception, design, data collection, data analysis, study supervision, writing, revising, and editing of the paper. Mohamud S. Mohamud and Emad M. Masuadi participated data cleaning, data analysis, and revising of the paper. Hassan S. Alamri participated in data analysis, revising, and editing the paper.

## ETHICS STATEMENT

Approval to perform this human participant research, with study number SP18/180/R and memo reference number IRBC/1696/18, was obtained from the Ethics Committee of the Institutional Review Board (IRB), King Abdullah International Medical Research Center (KAIMRC), Riyadh, Saudi Arabia. All procedures followed were in accordance with the ethical standards of the responsible Committee on Human Experimentation and with the Helsinki Declaration of 1975, as revised in 2000. The informed consent was provided by the IRB Office at KAIMRC.

## Supporting information


Appendix S1

## Data Availability

Data will be furnished by the corresponding author upon request.
